# HAZARDOUS WASTE: GAO Grades Hanford Cleanup

**DOI:** 10.1289/ehp.117-a539

**Published:** 2009-12

**Authors:** Harvey Black

**Affiliations:** **Harvey Black of Madison**, Wisconsin, has written for *EHP* since 1994 as well as for *Environmental Science & Technology*, *ChemMatters*, and the *Milwaukee Journal Sentinel.*

In 1989 the Berlin Wall came down. The same year, the U.S. Department of Energy (DOE) began a large-scale initiative to clean up the radioactive waste from creating plutonium for nuclear weapons at its Hanford Site in Washington. While the world has changed dramatically since the Wall came down, change has come more slowly to the Hanford Site. “Over the past 20 years, DOE has tried developing various approaches for treating and disposing of these wastes, at varying costs and with little success,” wrote the authors of a September 2009 Government Accountability Office (GAO) report titled *Nuclear Waste: Uncertainties and Questions about Costs and Risks Persist with DOE’s Tank Waste Cleanup Strategy at Hanford*.

Some 56 million gallons of radioactive waste from Hanford now sits at a storage site upriver from 200,000 people. The waste is stored in 177 underground tanks. Generally speaking, 98% of the radioactivity in the waste comes from strontium-90 and cesium-137, which have half-lives of about 30 years. The other radioactive components have half-lives stretching into the millions of years. There are also “large volumes of hazardous chemical waste . . . [that] can remain dangerous for thousands of years,” according to the report.

The DOE effort is governed by the Tri-Party Agreement, which describes the roles, responsibilities, and authority of the U.S. Environmental Protection Agency, the DOE, and the state of Washington. The agreement also establishes a plan to remove 99% of the waste from the tanks and sets milestones for completing certain tasks.

When the cleanup effort began, “it was not appreciated how hard it was going to be,” says Micah Lowenthal, a National Research Council staff member who worked on that group’s 2005 report *Risk and Decisions about Disposition of Transuranic and High-Level Radioactive Waste*. He points out, however, that some progress has in fact been made at the Hanford Site.

“They have spent billions of dollars on the tank wastes, and people might wish it were spent more efficiently, but it is not as though we haven’t gotten anything for our money,” he says. For instance, 149 of the 177 tanks are single-walled tanks, and 67 of these are known or suspected to have leaked. Lowenthal says the DOE has transferred the free liquids from all the single-walled tanks to the double-walled tanks. Furthermore, he says, the DOE has taken steps to deal with other environmental and safety emergencies on the site and stabilize the situation. “Now,” he says, “the agency is working on the long-term problem of retrieving, processing, and preparing the waste for disposal.”

The GAO report details a number of technical concerns about the Hanford cleanup effort, which involves analyzing the composition of the waste, retrieving it from the storage tanks, separating it into appropriate waste streams, vitrifying it (immobilizing it in glass), and placing it into stainless steel containers, which will be stored in a permanent repository. Each of these steps faces uncertainties. For instance, the system to collect and analyze the waste may not operate as quickly and efficiently as intended, substantially slowing the whole waste treatment process. Also, the system to transport waste from the tanks to a new waste treatment plant may clog and inadequately filter out certain parts of the waste. The report says DOE efforts to test this system using simulated waste “may not uncover all potential problems.”

The new plant is slated to start operating in 2019, but questions remain as to whether it will work as envisioned. Each step in the waste treatment process has to handle the chemical form of the waste it is fed. And the waste is very complex, says Gene Aloise, GAO director of natural resources and environment and lead author of the September report, who adds, “It’s unlike anything else in the world.”

And that complexity may translate into higher cleanup costs than anticipated. Cleanup could be complete as soon as 2042, but the GAO estimates the projected budget of $77 billion may balloon to $86–100 billion if the effort extends to 2054. “The cost has a lot of factors in it,” says Lowenthal. “In addition to the fact that they’re expected to do this unprecedented effort, they’re expected to do it with a level of worker safety . . . that is also unprecedented.”

The DOE is working to solve the problems identified by the GAO, says Erik Olds, director of communications of the department’s Office of River Protection, which manages tank waste cleanup at the Hanford Site. “We expect by either the end of this calendar year or early next year to have completed and closed all of those issues . . . that the GAO referenced in its report,” he says.

Although he talks optimistically, Olds acknowledges the DOE record at Hanford does not lend itself to optimism. “There is a history at the site of plans that were made for the treatment of tank waste that were never implemented for a variety of different reasons. One is that there were questions about the performance of the form, a grout, in which the waste would be immobilized,” he says. He adds that the capability of treating waste has evolved over the years and that the new waste treatment plant is over half built.

The report references a forthcoming environmental impact statement (EIS) that will be used as the basis for several decisions about Hanford’s cleanup, among them the final condition of the underground tanks, the final treatment and disposal of the wastes in those tanks, and whether waste from other DOE sites will be allowed to be stored there. According to the report, “the [EIS] provides an opportunity to use available risk assessment guidelines to consider scenarios the department has not considered to date—in particular, the possibility of removing varied quantities of waste from the tanks.” The DOE issued the draft EIS on 30 October 2009 in cooperation with the Washington State Department of Ecology. It will be open for public comment until 19 March 2010.

## Figures and Tables

**Figure:**
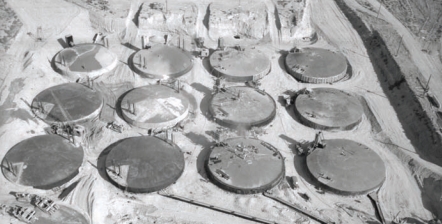
Underground storage tanks being built at the Hanford Site circa 1947.

